# Peripheral ossifying fibroma

**DOI:** 10.4103/0973-029X.80023

**Published:** 2011

**Authors:** Mahavir B Mishra, Kundendu Arya Bhishen, Shanu Mishra

**Affiliations:** *Department of Periodontology, Mahatma Gandhi Dental College and Hospital, Jaipur, Rajasthan, India*; 1 *Department of Oral and Maxillo-Facial Pathology, Mahatma Gandhi Dental College and Hospital, Jaipur, Rajasthan, India*; 2 *Rotherham General Hospital, Rotherham, Yorkshire, UK*

**Keywords:** Fibroblast, osteoid tissue, peripheral cemento-ossifying fibroma, peripheral ossifying fibroma, pyogenic granuloma

## Abstract

Here, we present a case report of peripheral ossifying fibroma (POF) in an adult lady in her fourth decade of life. This case report comprises the growth that occurred in the mandibular anterior region with displacement of anterior teeth, its satisfactory management and literature review. POF represents a reactive benign lesion of connective tissue and is not the soft tissue counterpart of ossifying fibroma and is also not related anyhow to peripheral odontogenic fibroma. POF in the age of 45 years, arising in the mandibular anterior region, is an occasional entity. Careful clinical examination and histopathology findings should be correlated to conclude the final diagnosis.

## INTRODUCTION

Benign fibrous overgrowths arising from the mucous membrane are termed as fibromas and are frequent growths in the oral cavity.

Many of the fibrous growths originate from underneath the periodontium, similar to peripheral ossifying fibroma (POF). POF is an occasional growth of the anterior region of mandible and accounts for 3.1% of all oral tumors and 9.6% of the gingival lesions. About 60% of these tumors occur in maxilla and more than 50% of all cases of maxillary POF are found in the incisors and canine areas. A female of 45 years of age had growth arising in the mandibular anterior region and it displaced the central, lateral incisors and canine. In this age group and in mandibular anterior quadrant, POF is very occasional and has not so far been reported in the literature.

## CASE REPORT

A non-smoker female, aged 45 years, reported with a moderately large growth in the mandibular anterior region. Medical history was insignificant. The patient was from low socioeconomic class. History revealed that a small nodule appeared 5 months ago, which was painless and caused spacing in the lower anterior teeth.

On examination, the patient’s lips were incompetent. Lower lip approximation was difficult due to large swelling protruding from the lower gingival region. Oral hygiene was considerably poor, which may be due to poor oral hygiene awareness, since oral hygiene is inversely related to socioeconomic class.

**Figure 1:**
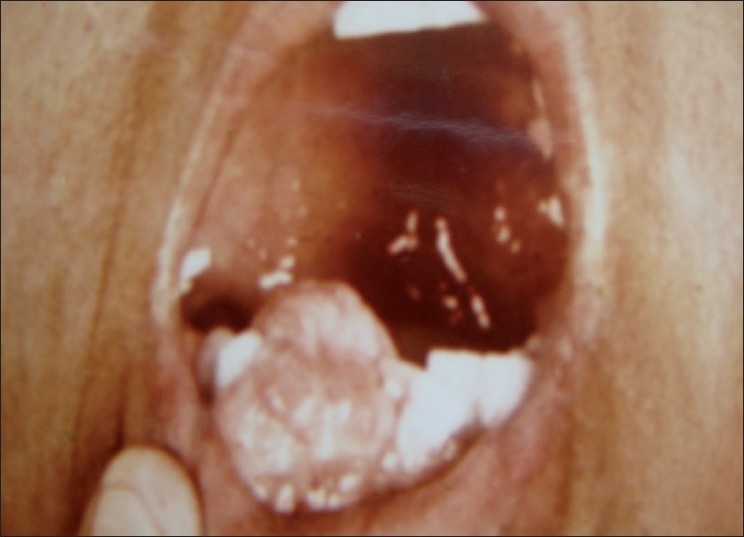
Female patient aged 45 years with POF in mandibular anterior region, displacing central, lateral and canine teeth

There was a pale pink swelling in the mandibular right central and lateral incisor region. The surface was nodular and irregular, with no ulceration. The growth measured 2.2 × 3.0 cm in size and was extending out from labial gingiva, displacing the central, lateral incisors and canine teeth, and extended lingually. The nodular growth extension in lingual was slightly smaller than that in the labial [Figure 1]. The growth was considerably hard in consistency, sessile and not easily movable.

Intraoral periapical radiograph was taken, which revealed erosion of the crest of bone. The possible reason of crestal bone erosion in the area may be long-standing plaque-induced inflammation and constant pressure of the growth. Clinical appearance and consistency was of a hard fibrous growth, which therefore led to a provisional diagnosis of peripheral calcifying fibroma or peripheral odontogenic fibroma (POdF).

Phase 1 periodontal treatment was carried out. Consent for the surgical procedure was obtained from the patient after proper counseling.

Under local anesthesia, the whole growth was excised and the underlying surface was thoroughly curetted up to deepest possible tissue and crestal osteoplasty was done. After controlling bleeding, periodontal dressing was applied and the patient was discharged with prescription of pain killer and chlorhexidine mouth wash. Follow-up visits were arranged after 1 week, followed by 1 month, 3 months, 6 months and 1 year and 2 years. Recall was necessary to rule out recurrence of the lesion, since it eroded alveolar bone and appeared aggressive.

**Figure 2:**
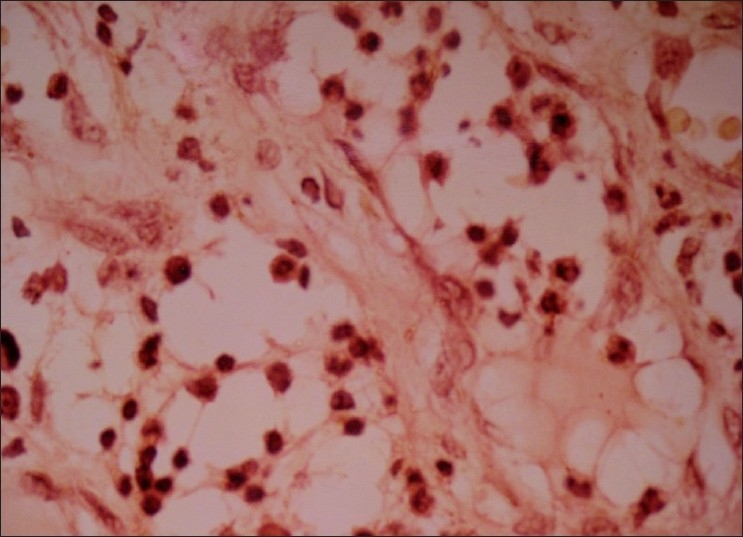
Microscopic picture of biopsy slide showing- proliferated fibrous bands in the connective tissue with uniformly distributed chronic inflammatory cells

**Figure 3:**
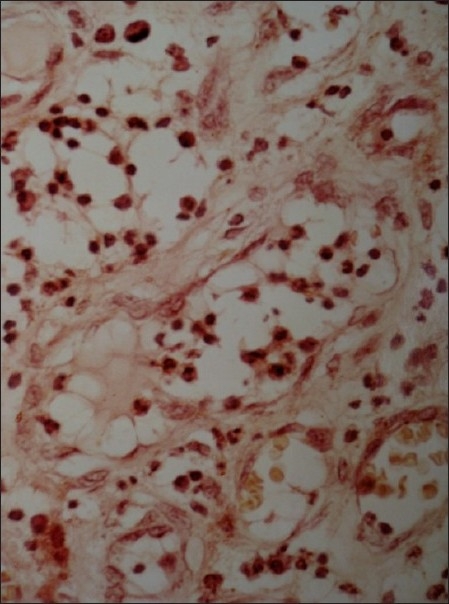
Micro-photograph of another specimen of the same biopsy, showing thick fibrous whirls

**Figure 4:**
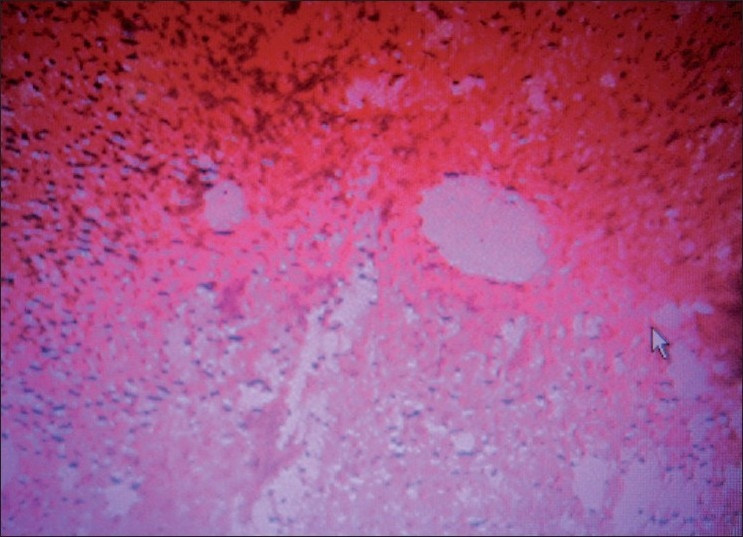
Multiple foci of osteoid tissue, surrounded by fibrous whirls

**Figure 5:**
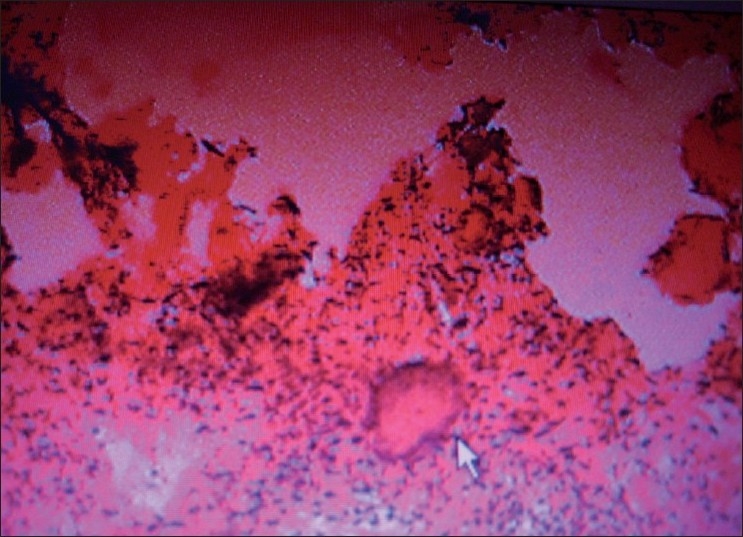
Sam slide at further location showing multiple foci of osteoid tissue, surrounded by fibrous bands

The growth was sent for the histo-pathologic evaluation. The report revealed an over growth of fibrous tissue. The connective tissue of the growth comprised of bundles of collagen fibers in a cellular stroma [Figure 2]. Numerous plumps to spindle shaped fibroblasts and fibrocytes were present. These cells were also arranged in a whirl shaped around irregular mineralization foci in the center [Figure 3]. Few blood vessels with RBC and proliferating endothelial cells were also evident. Chronic inflammatory cell infiltrate was seen evenly distributed in whole area and the cells comprised mainly of lymphocytes and plasma cells [Figure 4]. The calcified areas resembled cementum-like and bone-like ossifying areas [Figure 5].

Few blood vessels with RBC and proliferating endothelial cells were also evident. Chronic inflammatory cell infiltrate was seen evenly distributed in the whole area and the cells comprised mainly lymphocytes and plasma cells.

The overlying epithelium was hyperplastic parakeratotic stratified squamous epithelium showing numerous elongated rete ridges. Clinical assessment and histopathologic report confirmed and established the diagnosis as POF.

## DISCUSSION

In oral cavity periodontium can show different types of focal overgrowths. These lesions arise due to overgrowth and proliferation of different components of connective tissue in periodontium, i.e. the fibers, bone, cementum, blood vessel or any particular type of cell. The lexicon of focal proliferative lesions commonly occurring on gingival tissue includes fibroma, giant cell fibroma, pyogenic granuloma, peripheral giant cell granuloma, POF and POdF.[1] Most of these lesions are reactive chronic inflammatory hyperplasias, with minor trauma or chronic irritation being the etiologic factors.[2]

The nomenclature of these lesions is done in such a way so as to highlight the difference in nature of growth, location of growth, origin and the dominant proliferating histological component/cells in the lesion. The lesions which are present intraosseously are termed as “central lesions”, whereas their extraosseous counterparts or the lesions which appear on outer soft tissue (e.g. gingiva) are termed “peripheral lesions”. Also, a lesion may arise due to inflammation because of a stimulus and is called as a “reactive lesion” or it can be truly “neoplastic” where it is classified as a benign or a malignant neoplasm.

The term POF was coined by Eversole and Robin.[3] It occurs exclusively on gingiva. It is a relatively common growth of gingiva and is considered to be reactive in nature rather than neoplastic.[4] POF is characterized by a high degree of cellularity usually exhibiting bone formation, although occasionally, cementum-like material or dystrophic calcification may also be found.[1]

As the lesion occurs only on gingiva and is supposed to be derived from periodontal ligaments, some authorities believed the lesion to be odontogenic in origin.[1]

Presently, the origin and pathogenesis of the lesion is unknown. However, due to their clinical and histopathologic similarity, it is considered that at least some cases of POF may arise as a result of maturation of a long-standing pyogenic granuloma.[4]

The mineralized product in POF probably has its origin from cells of periosteum or periodontal ligament.[5]

The POF has a peak incidence in young and teenaged females.[4] Cundiff reported that the lesion is prevalent between ages of 5 and 25 years, with a peak incidence at 13 years of age. Cundiff also reported a definite female predilection.[6] Female to male ratio may vary from 2:1 to 3:2. The site of occurrence of POF is usually anterior to molars in both maxilla and mandible equally,[1] and in more than 50% of cases in the incisor, and cuspid regions.[5]

Clinically, the lesion appears as a nodular mass which may be pedunculated or sessile, pink to red in color and surface is usually but not always ulcerated. In the present case also, the lesion occurred in a middle-aged female in mandibular anterior region and appeared as a nodular pale to pink growth without ulceration.

Histopathologically, the lesion shows stratified squamous epithelium covering an exceedingly cellular mass of connective tissue made up of plump fibroblasts, fibrocytes, fibrillar stroma and areas of mineralization with multinucleated giant cells near them in some cases. The mineralization may consist of bone, cementum-like material or dystrophic calcifications. The dystrophic calcifications are usually seen in early, ulcerated lesions, whereas the older, mature, non-ulcerated lesions show well-formed bone and cementum-like material,[5] as was evident in the present case also.

The clinical differential diagnosis of POF includes all the nodular lesions which occur on gingiva (as mentioned before). Histopathologically, it is very important to understand the difference between the similar sounding lesions, i.e. POF, POdF, central ossifying fibroma (COF) and central odontogenic fibroma (COdF).

Despite of similarity in terminology, POF is a completely distinct entity from POdF and COF. The POF, representing a reactive benign lesion of connective tissue, is not the soft tissue counterpart (or related anyhow) to central ossifying fibroma which represents an osteogenic neoplasm.[5]

Similarly, the reactive lesion POF is completely different from POdF which is in fact a neoplasm of odontogenic ectomesenchyme with included odontogenic epithelium. As opposite to POF and COF, the POdF does represent the extraosseous counterpart of the COdF and is related to it [Table 1].

**Table 1:** The important differences between POF, COF, POdF and COdF

POdF	POF	COF	COdF
Classified under odontogenic tumors of ectomesenchyme with or without included epithelium (Neoplasm)	Classified under benign connective tissue lesions and may arise due to inflammation (Reactive)	Classified under fibro-osseous lesion and represents an osteogenic tumor (Neoplasm)	Classified under odontogenic tumors of ectomesenchyme with or without included epithelium (Neoplasm)
Rare lesion of gingiva	Common lesion occurring only on gingiva	Common lesion in long bones but rare in skull and jaw bones	Very rare lesion occurring in jaw bones
It is a soft tissue (extraosseous) counterpart of COdF	It does not represent a soft tissue (extraosseous) counterpart of COF	Present centrally (intraosseously) – a distinct lesion from POF	Present centrally (intraosseously) – related to POdF
Histologically same as WHO type of COdF in gingiva	No further types or subclassifications	It is of two types: 1) Psammomatoid 2) Juvenile type	It is of two types: 1) Simple type (with no mineralization) 2) WHO type (with bone/ cementum)

POF - peripheral ossifying fibroma; COF - counterpart of ossifying fibroma; POdF - peripheral odontogenic fibroma; COdF - central odontogenic fibroma

## CONCLUSION

The POF represents a reactive benign lesion of connective tissue and is not the soft tissue counterpart of ossifying fibroma (COF) and is also not related anyhow to POdF. It occurs frequently in anterior part of jaws of young females, exclusively on gingiva. The accepted treatment protocol includes surgical excision followed by histopathologic evaluation and follow-up.
